# Novel Angiotensin-I Converting Enzyme Inhibitory Peptides Isolated From Rice Wine Lees: Purification, Characterization, and Structure-Activity Relationship

**DOI:** 10.3389/fnut.2021.746113

**Published:** 2021-09-10

**Authors:** Zeqi He, Guo Liu, Zijiao Qiao, Yong Cao, Mingyue Song

**Affiliations:** Guangdong Provincial Key Laboratory of Nutraceuticals and Functional Foods, College of Food Sciences, South China Agricultural University, Guangzhou, China

**Keywords:** hypertension, angiotensin-I converting enzyme, rice wine lees, ACE-inhibitory peptides, structure-activity

## Abstract

The bioactive peptides that can inhibit angiotensin-I converting enzyme (ACE, EC. 3. 4.15.1) are considered as possible cures of hypertension. Food-derived angiotensin-I converting enzyme inhibitory (ACEi) peptides have gained more attention because of their reduced side effects. In this study, we reported the method for purifying ACEi peptides from the lees of traditional Chinese rice wine and evaluated the product's biochemical properties. After three steps of reversed-phase high-performance liquid chromatography (RP-HPLC), for the first time, we isolated, purified, and identified two novel peptides: LIIPQH and LIIPEH, both of which showed strong ACEi activity (IC_50_-values of 120.10 ± 9.31 and 60.49±5.78 μg/ml, respectively). They were further categorized as mixed-type ACE inhibitors and were stable against both ACE and gastrointestinal enzymes during *in vitro* digestion. Together, these results suggest that the rice wine lees that produced as a by-product during rice wine production can be utilized in various fields related to functional foods and antihypertensive medicine.

## Introduction

Raised blood pressure is a major cardiovascular risk factor (1). As one of the leading risk factors for global mortality, uncontrolled hypertension causes stroke, myocardial infarction, cardiac failure, dementia, and other diseases (2). According to the World Health Organization (WHO), hypertension causes an estimated 9.4 million deaths yearly and represents 7% of the global disease burden (3). Once hypertension develops, lifelong pharmaceutical treatment is typically required. Several types of antihypertensive drugs have been developed based on different therapeutic targets, including angiotensin-I converting enzyme (ACE) inhibitors (4). Angiotensin-I converting enzyme (dipeptidyl carboxypeptidase, EC 3.4.15.1) is a zinc metallopeptidase that is found in male genital, vascular endothelial, neuro-epithelial, and absorptive epithelial cells (5). Angiotensin-I converting enzyme plays dual roles in the renin-angiotensin system (RAS) to regulate blood pressure by degrading angiotensin-I (Ang I) into angiotensin-II (Ang II), a strong vasopressor. Moreover, in the kallikrein-kinin system (KKS), ACE triggers inactivation of bradykinin, an important vasodilator (6, 7). Currently, ACE inhibitors are considered to be the first-line treatment for hypertension (8). Compared to other regimens, treatment regimens based on ACE inhibitors have been shown to reduce risk of coronary artery disease, cardiovascular death, and heart failure, and these treatments are frequently used for this reason (7, 9). However, the long-term administration of several synthetic ACE-inhibition drugs, such as captopril, enalapril, and lisinopril, is still associated with undesirable side effects, including headache, dry cough, and edema (9–11). Furthermore, drug treatment can be costly, and the high prevalence of hypertension makes it a challenge for resource-constrained settings (3). Therefore, the development of safe, cost-effective, and efficient treatment approaches is urgently needed. Angiotensin-I converting enzyme inhibitory peptides derived from food sources represent an effective way to treat hypertension without incurring unacceptable side effects (12). Hence, much interest has been focused on the extraction of ACE inhibitors from natural food sources as a replacement for synthetic drugs (13).

Numerous protein-based foods, including milk (14, 15), eggs (8), fish (16, 17), and rice (18–20), have been used to prepare angiotensin-I converting enzyme inhibitory (ACEi) peptides through enzymatic hydrolysis. Rice (*Oryzae sativa*, Family: Oryzeae) is a major cereal crop worldwide (21). It is a dietary staple for about half of the world's population and is particularly common in Asian countries, where it serves as a substantial source of dietary protein for millions of people (21–23). Compared to those of other cereals, rice proteins have a preferable amino acid structure due to their essential amino acid composition (24). In addition, the high availability of processing by-products has led rice to attract even much attention as a potential source of biologically active hydrolysates and peptides (25). Wine lees is the main by-product of rice wine production, and more than 400,000 tons are generated annually. Wine lees are composed of solid and liquid fractions whose compositions depend on their regions of origin and the associated agronomic and edaphoclimatic characteristics (26). The protein content of wine lees is approximately 28.0% (dry basis), making it a good protein resource.

Six novel ACEi peptides, AVQ, YPQ, NQL, AYLQ, VLPVLS, and VLPSLN, have successfully been identified from solid fractions of wine lees (27). However, due to the high moisture and organic acid contents, the liquid fraction of wine less has received comparatively less attention. As a product of rice fermentation, the liquid phase of lees contains considerable amounts of peptides and proteins. Therefore, it is urgent to development the comprehensive utilization of the liquid phase of rice wine lees, as it has the potential to provide new ideas for the treatment of rice wine lees to reduce environmental pressure.

In this study, we purified and identified ACEi peptides from rice wine waste using RP-HPLC and MOLDI-TOF-MASS. The IC_50_-values of the products were determined, and their inhibition types were kinetically characterized using a Lineweaver–Burk plot. Additionally, we investigated the stability of these compounds against digestive proteases and ACE. In doing so, this work contributes to the comprehensive utilization of rice wine waste and provides a theoretical basis for the development of effective antihypertensive peptides.

## Materials and Methods

### Materials and Chemicals

Rice wine lees was obtained from Guangdong Shunde Winery Co., Ltd., Shunde, China. Angiotensin-I converting enzyme (from rabbit lung) and N-Hippuryl-His-Leu hydrate (HHL) were purchased from Sigma Co., USA. Other chemicals and reagents were of analytical grade.

### Physicochemical Characterization of Rice Wine Lees Extract

To prepare Rice Wine Lees Extract (RWLE) for characterization, large particles were removed by centrifuging at 4,000 rpm for 10 min, after which the supernatant was concentrated and lyophilized. The molecular weight distribution of RWLE was determined as previously described (28) with some modifications. Specifically, the molecular weight distribution was determined via gel permeation chromatography (GPC) with a TSK gel filtration column, G2000 SWXL (300 × 7.8 mm; Tosoh, Tokyo, Japan). The mobile phase, water/acetonitrile/trifluoroacetic acid (80/20/0.1, v/v/v), was delivered at a flow rate of 0.5 ml/min. The determination was carried out at room temperature, and the UV detector was operated at 220 nm. A calibration curve was obtained using the following standards: insulin (5,808 Da), bacitracin (1422 Da), tetrapeptide GGYR (451 Da), and tripeptide GGG (189 Da). Then, a molecular weight calibration curve (*Y* = −0.336*X* + 9.043, *R*^2^ =0.993) was obtained. In addition, fractions from different molecular weights were collected, concentrated, and tested using the ACE inhibition assay described below.

### Measurement of ACEi Activity

Angiotensin-I converting enzyme inhibitory activity was measured using the method described in Wu et al. (29) with some modifications. For each reaction, 10 μl of 0.25 U/ ml ACE solution (prepared in 0.01 M potassium phosphate buffer containing 0.5 M NaCl, pH 7.0) was mixed with 10 μl of inhibitor solution and incubated for 5 min at 37°C. Subsequently, 30 μl of HHL solution (5 mM in 0.1 M sodium borate buffer containing 0.3 M NaCl, pH 8.3) was added, followed by incubation at 37°C for 1 h. The reaction was terminated with the addition of 70 μl 1 M HCl before subsequent assays were performed. For the control group, 10 μl ACE solution and 80 μl 1 M HCl were simultaneously added to the mixture. A blank sample was prepared by replacing the inhibitor solution with 10 μl borate buffer. Samples were then separated using a C18 column (4.6 × 150 mm, 5 μm) at a flow rate of 1.0 ml/min. The mobile phase was water/acetonitrile/trifluoroacetic acid (75/25/0.1, v/v/v) with an isocratic gradient of 25% B in 15 min. Absorbance was monitored at 228 nm. Angiotensin-I converting enzyme activity (%) was calculated according to the following equation:


$$\begin{array}{l} ACE\;inhibition\left(\%\right)\;=\;\left(A1-A2\right)-B/\left(A1-A2\right)\times 100\% \end{array}$$

Where A1, A2, and B are peak areas corresponding to Hippuric Acid in the control group, the blank and the sample, respectively. The IC_50_-value was defined as the concentration of inhibitor required to inhibit 50% of enzyme activity and was calculated by performing regression analysis of ACE inhibition vs. sample concentration.

### Purification of ACEi Peptides

Angiotensin-I converting enzyme inhibitory peptides were first isolated by preparative high-performance liquid chromatography (LC-8, Shimadzu, Japan) using a reversed-phase (RP) column [Shimadzu PRC-ODS(K) 30 × 250 mm, 15 μm] of C18. Solvent A was 0.1% trifluoroacetic acid in double-distilled water, and solvent B was 0.1% trifluoroacetic acid in 100% acetonitrile. A linear gradient of solvent B was applied at a flow rate of 10 ml/min for 55 min as follows: 10–34% solvent B for 30 min and 30–45% solvent B for 45 min. Fractions were collected, concentrated, and tested using an ACE-inhibition assay. The fraction with the highest ACEi activity was further separated using an additional C18 column (ECOSIL 300 × 20 mm,10 μm, Germany) and the same mobile phases. A linear gradient of solvent B was applied at a flow rate of 10 ml/min for 45 min as follows: 15–19% solvent B for 5 min and 19–23% solvent B for 45 min. To further purify the ACE inhibitor, active fractions were analyzed using HPLC to identify monomeric peptides with increased ACEi activity. The active fraction solution collected previously was filtered through a 0.45 μm membrane filter and separated using an ECOSIL C18 column (260 × 4.6 mm, 5 μm). Mobile phases were solvent A (double-distilled water with 0.1% trifluoroacetic acid) and solvent B (acetonitrile with 0.1% trifluoroacetic acid). Elution was performed using water/acetonitrile/trifluoroacetic acid (80/20/0.1, v/v/v) with 5–15% solvent B (0–10 min) and 15–20% solvent B (10–45 min) at a flow rate of 1 ml/min. Main peaks were collected, concentrated, and lyophilized. All elution steps were monitored at 214 nm. The ACEi activity of the eluted peaks was tested.

### Amino Acid Sequencing of Purified Peptides

The amino acid sequences of active peptides displaying ACEi activity were analyzed using a matrix-assisted laser desorption ionization time-of-flight mass spectrophotometer (MALDI-TOF-MASS, Bruker Daltonik GmbH, Bremen, Germany).

Peptide recognition was performed using an automated protein/peptide sequencer (model PPSQ-53A, Shimadzu, Tokyo, Japan). The purified peptide was dissolved in deionized water and transferred onto a polyvinylidene fluoride membrane. After fixation with polybrene, the amino acid sequence of the peptide was characterized.

### Synthesis of Identified Peptides

The sequences of the identified peptides were submitted to Synpeptide Co., Ltd. (Shanghai, China) for peptides synthesis using solid-phase methods. Purity and molecular weights of synthesized peptides were determined using HPLC and LC-MS.

### Stability of Purified Peptides During *In Vitro* Digestion by Gastrointestinal Enzymes

Peptide stability was measured according to the methods described in Chen et al. (30) with some modifications. For this assay, 2% (w/w) pepsin was dissolved in a KCl–HCl buffer (0.1 mM) adjusted to pH 2.0, and 2% (w/w) chymotrypsin was dissolved in a KCl–NaOH (0.1 mM) buffer adjusted to pH 7.0. Peptides were dissolved at 1 mg/ml in pepsin or chymotrypsin solutions and incubated at 37°C for 4 h. Reactions were terminated by boiling for 15 min. After centrifugation at 4,000 rpm for 15 min, the supernatant was collected, pH was adjusted to 8.0, and samples were assayed for ACEi activity. The supernatant was also analyzed using the previously described RP-HPLC conditions to evaluate changes to the peptides after *in vitro* digestion.

Control groups were prepared by incubating inactivated pepsin and/or chymotrypsin (boiled for 15 min) with the sample peptides. After incubation, the mixture was processed and analyzed as described.

### Stability of Peptides Against ACE

The stability of peptides against ACE was assayed according to the methods of Deng (31), with some modifications. For each reaction, 30 μl of peptides (2 mg/ml) was combined with 30 μl 0.25 U/ml ACE solution and incubated at 37°C for 30 min. Angiotensin-I converting enzyme was then inactivated by boiling the solution for 10 min. As described in section Measurement of ACEi Activity, 20 μl of each peptide sample was used for the detection of ACEi activity.

### Kinetic Parameters of ACEi Peptides

Angiotensin-I converting enzyme inhibition of purified peptides was determined using Lineweaver–Burk plots according to reported methods (32). Samples were added at two fixed concentrations to each reaction mixture, and enzyme activity was measured for different concentrations of the substrate HHL according to the ACE inhibition assay. The curves were described by 1/area and 1/[S], and the kinetics of ACE inhibitors were estimated by comparing the resulting curves.

### Design of ACEi Peptides According to Source Proteins

As protein structure has a significant impact on ACE-inhibition (33–35), we chose to prune or add peptides according to UNIPROT (https://sparql.uniprot.org/) BLAST results. The newly designed peptides were synthesized and further assessed for ACE-inhibition capacity as described above.

### Statistical Analysis

Analysis of variance was performed with SPSS 13.0 software (Armonk, NY, USA). Linear regression was performed using Origin (Microsoft Corp., Seattle, WA, USA).

All experiments were repeated at least three times, and mean values were used. Data were subjected to analysis of variance, and Duncan values were calculated with a confidence interval of 95% to compare means.

## Results and Discussion

### Physicochemical Properties and ACEi Activity of Rice Wine Lees Extract

Rice Wine Lees Extract was prepared as described and found to exhibit potent ACEi activity, with an IC_50_-value of 806.67 ± 58.97 μg/ml. Previously, wine and wine lees have been subjected to recovery or transformation of components into high value-added compounds (26).

**Figure 1:**
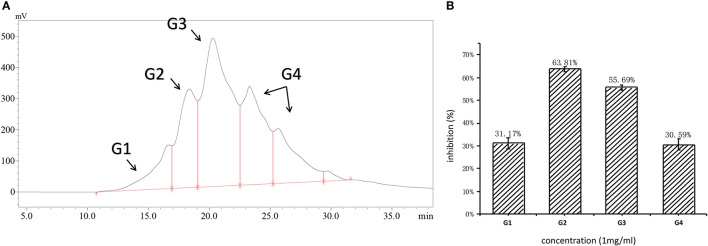
**(A)** Molecular distribution of RWLE; **(B)** inhibition of different molecular fractions (G1–G4).

The molecular weight distribution of RWLE was broad, ranging from 50 to 25,000 Da, indicating that it contained proteins and free amino acids (Table 1). Accordingly, RWLE was further divided into four components, G1–G4 (Figure 1A). As shown by the associated ratios, the 50–500 Da molecular weight segment accounted for a significant proportion of RWLE (37.89%).

**Table 1:** Molecular distribution of RWLE.

**MW (Da)**	**Distribution (%)**
3k−25k	10.19
500–3k	18.68
50–500	37.89
<50	33.24

Fractions G1–G4 were collected individually, and their ACEi activities were further determined. As shown in Figure 1B, G2 possessed the highest ACEi capacity at a concentration of 1 mg/ml. Therefore, we predicted that the molecular weights of ACEi peptides would fall in the range of 500–3,000 Da. This prediction is consistent with previous reports that antihypertensive peptides are typically short, with molecular weights >2,000 Da (36). Larger peptides are not capable to contact the ACE active site easily, resulting in reduced inhibitory capacity.

### ACEi Peptide Purification

**Figure 2:**
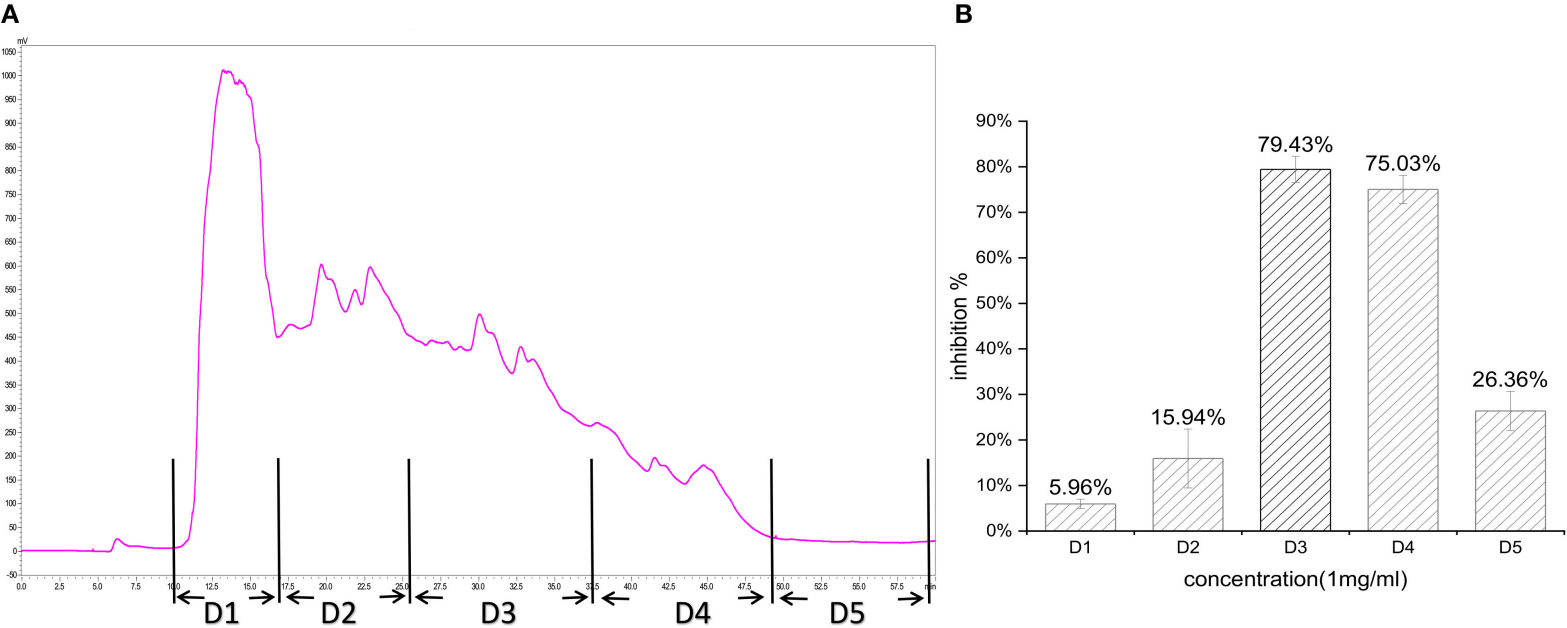
**(A)** Reverse-phase HPLC chromatogram of first-step purification of RWLE peptides; **(B)** inhibitory activity of five pooled fractions (D1–D5).

**Figure 3:**
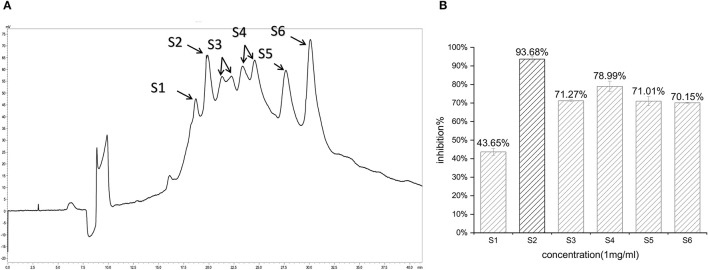
**(A)** Reverse-phase HPLC chromatogram of second-step purification of the G3 fraction; **(B)** inhibitory activity of six pooled fractions (S1–S6).

**Figure 4:**
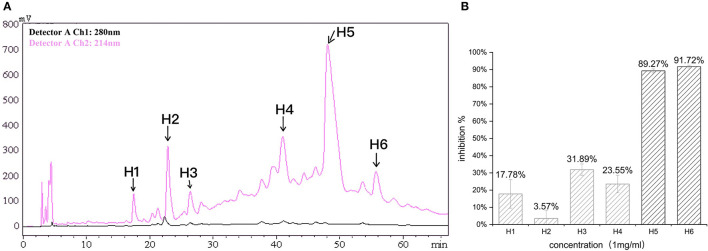
**(A)** Reverse-phase HPLC chromatogram of third-step purification of the S2 fraction; **(B)** inhibitory activity of six fractions (H1–H6).

In the first step, fractions between 20 and 55 min in the RP-HPLC chromatogram were collected and termed D1–D5 (Figure 2A). Fraction D3 showed the lowest IC_50_-value (643.33 ± 17.64 μg/ml) and was further separated into different fractions (Figure 2B). The seven main fractions were designated as S1–S7 (Figure 3A). Each fraction was subsequently collected. All of them exhibited ACEi activity at a concentration of 1 mg/ml, and the inhibition rate was in the range of 43.65–93.68% (Figure 3B). Importantly, activity analysis showed that Fraction S2 had the lowest IC_50_ (479.00 ± 59.36 μg/ml). Thus, this fraction was further purified by RP-HPLC using an analytical C18 column. As shown in Figure 4A, S2 was subsequently separated into six peaks (H1–H6). Among them, H5 and H6 were the two most potent ones in ACE inhibition with IC_50_-value of 120.10 ± 9.31 and 60.49 ± 5.78 μg/ml, respectively (Figure 4B). Accordingly, they were subjected to MOLDI-TOF-MASS to identify their amino acid sequence.

### Identification of Amino Acid Sequence of Purified ACEi Peptides

**Figure 5:**
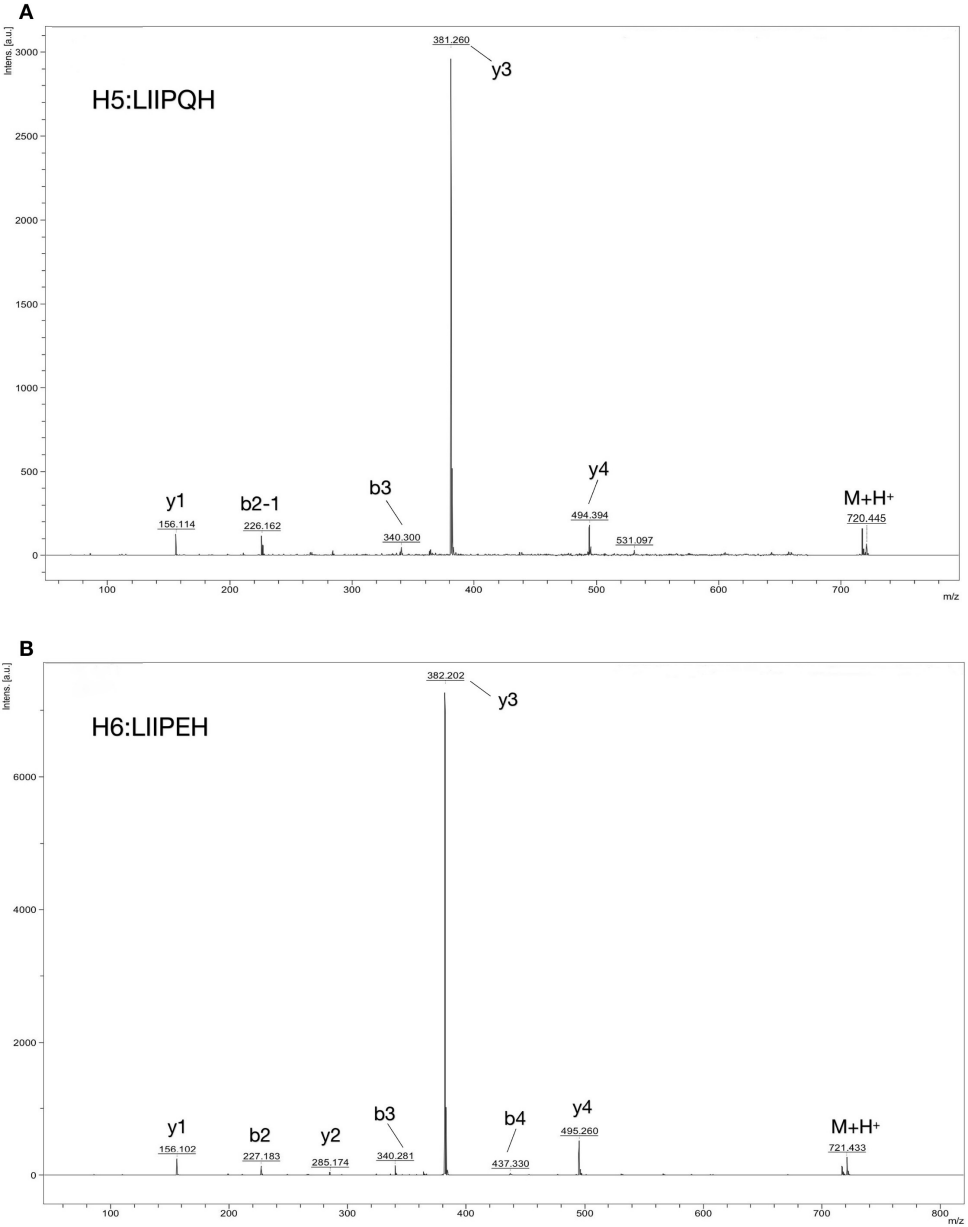
Identification of ACE-inhibition peptides from RWLE using MOLDI-TOF-MS. **(A)** Molecular mass and amino acid sequence of peptide H5; **(B)** molecular mass and amino acid sequence of peptide H6.

**Figure 6:**
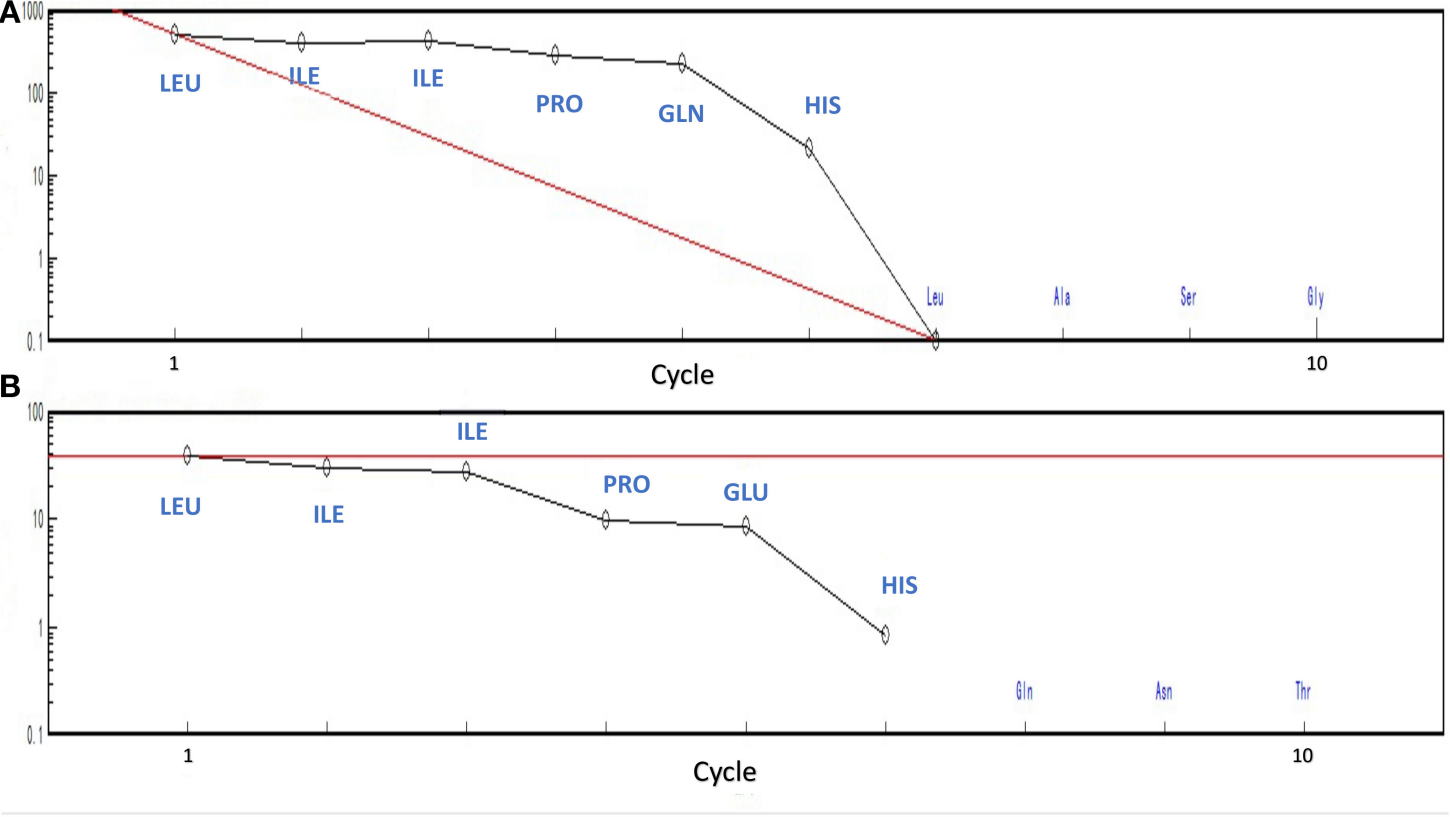
Identification of peptide's sequence using PPSQ **(A)** peptide H5 and **(B)** peptide H6.

MOLDI-TOF-MS/MS was used to determine the amino acid sequence of the peptides (Figure 5). H5 was found to have a molecular mass of 719.85 Da, and H6 had a molecular mass of 720.85 Da. Because Leu and Ile have the same molecular weight, we used an automated protein/peptide sequencer to further validate the peptides sequence. The results showed that H5 had a sequence of Ile-Leu-Leu-Pro-Gln-His, while H6 had a sequence of Ile-Leu-Leu-Pro-Glu-His (Figure 6). Markedly, it is the first time to isolate and identify these two novel peptides from food proteins. Additionally, their molecular weights correspond to the aforementioned results that the most active ACEi peptide was found to fall within the molecular weight range of <2,000 Da.

Interestingly, these two novel peptides are all oligopeptides with six amino acid residues and shared the sequence LIIP. It has been previously reported that most ACEi peptides are short, containing 2–12 amino acids (37). Peptides with hydrophobic amino acids, especially those with aliphatic chains such as Gly, Ile, and Leu at the N-terminus, have very high ACEi activity (38–40). Moreover, the presence of Leu at the final position has been claimed to enhance the ACE-inhibiting effects of an amino acid sequence (41, 42).

The C-domain of ACE is responsible for generating Ang II from Ang I under physiological conditions (43). Of note, the C-domain catalytic site of ACE consists of three subsites—S1, S1′, and S2′–which accommodate the three hydrophobic C-terminal residues of its natural substrate, angiotensin I (44). Hence, preferred ACE inhibitors contain hydrophobic (aromatic or branched side chains) amino acid residues at each of the three C-terminal positions (39). Based on studies further exploring ACEi penta- and hexapeptides and their structure-activity relationships, some rules have been concluded. These peptides contain, at minimum, two highly hydrophobic amino acid residues (8). Notably, the sequences of RWLE-derived ACE-inhibition peptides are consistent with these relationships between the primary structures of ACEi peptides and their inhibitory effects.

However, comparing the primary structures, the second N-terminal amino acids of H5 and H6 are different. Specifically, H5 contains Gln, an amino acid with a negative charge, while H6 contains Glu, a neutral amino acid. Although the role of positive charge at an amino acid remains unknown, it has been suggested that the presence of positively charged amino acids in the C-terminal region of the peptide could contribute to ACE-I activity and substantially increase inhibitory potency (45). This phenomenon might be explained by the presence of negatively charged Glu403 and Glu162 residues at the S2 and S1′ subsites of the sACE C-domain, respectively (43). The preference for positive charges at these two subsites might enhance selectivity to the sACE C-domain over the N-domain, therefor weakening ACE's ability to produce ANG II (46, 47). In theory, glutamic acid could act an important contributor to ACE inhibition by chelating zinc at the ACE active center and through hydrophobic interactions with ACE (48), which might be another reason for the differing IC_50_-values. Furthermore, the binding affinity of long peptides to ACE is influenced by the structure adopted in the specific environment of the binding site (49). Therefore, conformational changes in the peptide backbone can be induced by the polarity of these two residues and influence ACEi capacity (50).

In this work, the IC_50_-values of synthetic H5, H6, and the products in each purification step are summarized in Table 2. We found that synthetic peptides have no significant differences in comparison to natural peptides. Since limited amounts of natural peptides could be isolated from RWLE, synthetic peptides were used to further study the inhibition patterns and assess biological stability against ACE and gastrointestinal enzymes.

**Table 2:** IC_50_-values of the products in each purification step.

**Inhibitor**	**IC_**50**_ (μg/ml)**
RWLE	806.67 ± 58.97
G3	643.33 ± 17.64
S2	479.00 ± 59.36
H5	120.10 ± 9.31
H6	60.49 ± 5.78
H5 (synthesis)	143.33 ± 5.77
H6 (synthesis)	56.67 ± 12.02

### Stability of the Purified Peptides Against Gastrointestinal Enzymes

In contrast to chemically synthesized low-molecular-weight compounds, peptides are easily inactivated by gastrointestinal enzymatic digestion (51). In addition to demonstrating high inhibitory activity, purified peptides must remain sufficiently bioavailability during hydrolysis by gastrointestinal proteases after oral administration to bind their target sites and exhibit an antihypertensive activity *in vivo* (52).

**Figure 7:**
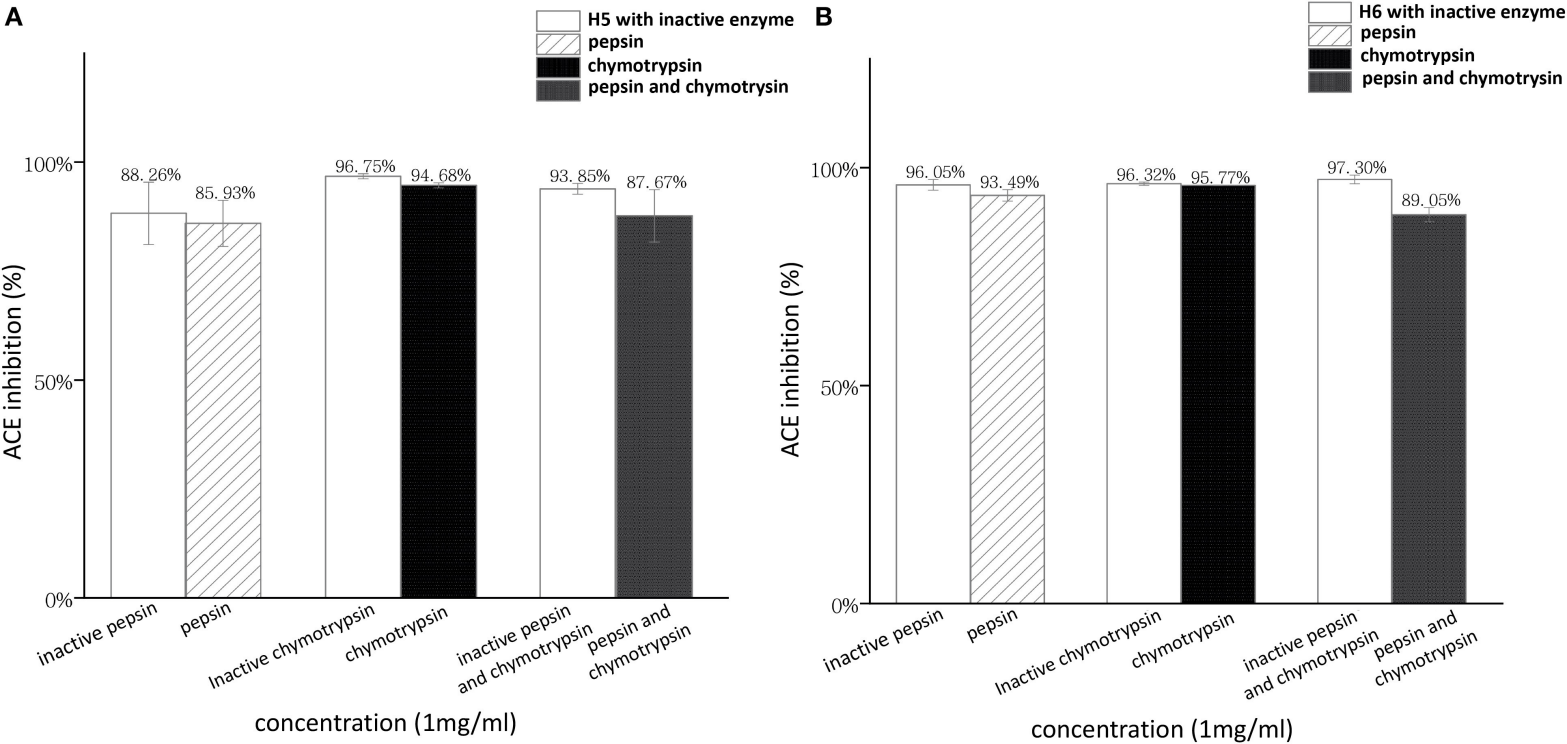
Stability of RWLE peptides against gastrointestinal enzymes **(A)** peptide H5 and **(B)** peptide H6.

In this work, LIIPQH and LIIPEH were subjected to *in vitro* digestion by pepsin and chymotrypsin (Figure 7). Although ACEi capacity of H5 and H6 was decreased by the combination of two gastrointestinal enzymes, the differences were not statistically significant (*p* > 0.05), suggesting that the peptides were stable within the gastrointestinal environment and could maintain their activities after being absorbed. Proline- and hydroxyproline-containing peptides are generally resistant to degradation by digestive enzymes (41, 53), so the great stability of these two peptides might be a result of a proline residue found in the third C-terminal position.

### Enzymatic Studies of ACEi Peptides

Due to the broad substrate specificity of ACE, some ACEi peptides may become ACE substrates and be subsequently cleaved into smaller fragments (54). Accordingly, ACEi peptides can be classified into three categories based on the results of ACE-ACEi peptides preincubation testing. The first type is true inhibitors, the second type is real substrates, and the third type is the pro-drug type (7). The stability of ACEi peptides we purified against ACE were summarized in Table 3, showing that both LIIPQH and LIIPEH had lower IC_50_-values compared to the control (co-incubation with inactive ACE). Therefore, these peptides could be categorized as pro-drug ACE-inhibitors, which showed improved ACEi activity due to the formation of a more active fragment after hydrolysis by ACE.

**Table 3:** Stability of RWLE peptides toward ACE.

**Peptide**	**IC_**50**_ with**	**IC_**50**_ without**
	**pre-incubation (mg/ml)**	**pre-incubation (mg/ml)**
LIIPQH	82.0 ± 19.92	65.88 ± 18.42
LIIPEH	51.0 ± 7.51	36.98 ± 21.08

**Figure 8:**
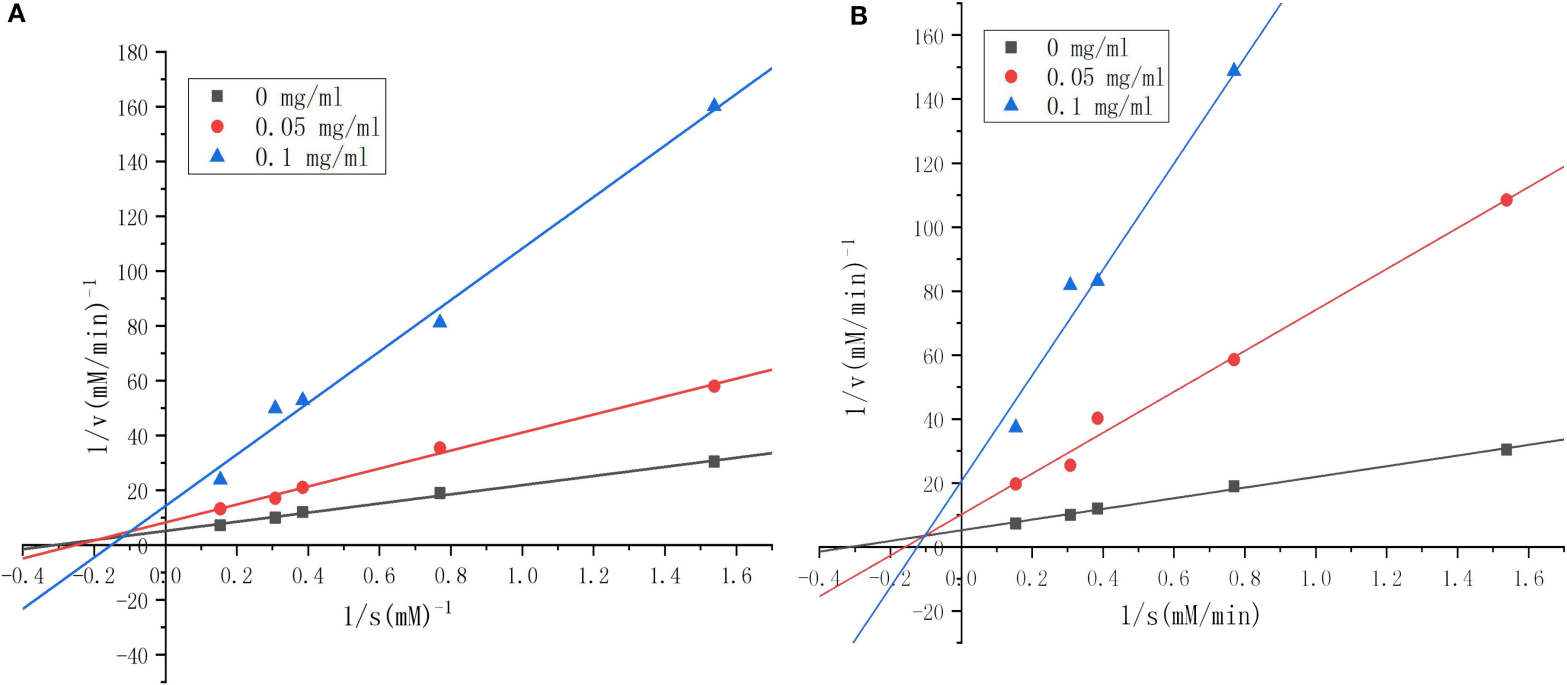
Lineweaver–Burk plots of angiotensin I-converting enzyme (ACE) inhibition by different concentrations of synthesized RWLE peptides across varying substrate concentrations (0–0.1 mg/ml). **(A)** Lineweaver–Burk plots of peptide H5 and **(B)** Lineweaver–Burk plots of peptide H6. V = initial rate of reaction [ΔA_228_ (nm)/min].

To clarify the kinetics of inhibition, Lineweaver–Burk plots were generated. Various concentrations of substrate were incubated with ACE solution in the absence or presence of RWLE peptides at 0.05 or 0.1 mg/ml. The results were illustrated in Figure 8A (H5) and Figure 8B (H6). We found that all lines intersected at similar x-intercepts, but had different slopes and y-intercepts, suggesting that H5 and H6 acted as non-competitive inhibitors (mostly mixed-type) toward ACE. Thus, H5 and H6 could bind to both free enzymes and the enzyme-substrate complex to reduce catalysis (55).

As Km and Vm are important parameters of enzymatic reactions, we further determined these values for the novel ACEi peptides. Vmax for the uninhibited ACE reaction was 0.191 min^−1^.Though Vm values decreased as peptide concentration increased for both H5 and H6, H6 appeared to be more effective (Table 4). The finding of a lower Vmax in the presence of H6 was consistent with its lower IC_50_-value compared to H5. Together, these results suggested that the activation energy of the catalytic reaction increased in the presence of peptides because of the decreased enzyme reaction rate. We hypothesized that the peptides blocked the substrate from binding to the ACE active site (56), resulting in the reduction of enzymatic efficiency. Based on a linear regression fit of the kinetic data, the Km-value of ACE activity in the absence of inhibiting peptide was estimated as 3.199 (Table 4). The Km-values increased when ACEi peptide was added to the reaction, suggesting that a higher concentration of substrate was required for the ACE catalytic reaction. The catalytic efficiency (CE) of the uninhibited ACE-catalyzed reaction was 0.060. In the presence of H5 or H6, it was decreased in a dose-dependent manner. The reduction of CE was directly related to the lower Vmax and IC_50_-values, suggesting that the peptides had binding affinity toward the target enzyme (7).

**Table 4:** Kinetic constants of ACE-catalyzed reactions at different peptide concentrations.

**Catalytic parameters**	**Control**	**H5**		**H6**	
Concentration (mg/ml)	0	0.05	0.1	0.05	0.1
Vm (ΔA/min)	0.191	0.120	0.070	0.099	0.049
Km (mg)	3.199	3.948	6.552	6.308	8.054
CE	0.060	0.030	0.011	0.016	0.006

*Km is the Michaelis constant, Vmax is maximum reaction velocity, CE is the catalytic efficiency (Vmax/Km)*.

The classification of “mixed-type inhibition” indicates that the peptide binds to ACE at both active and non-active sites, consequently reducing ACE's catalytic activity (57). As the space within the ACE active site is limited, which is hard for large peptides to bind. Therefore, peptides H5 and H6 may preferentially bind to the non-active site of ACE (57). Some research has explored mixed-competitive ACE inhibitors, including NMAINPSKENLCSTFCK and EKVNELSK isolated from casein (56, 57) and the synthesized peptides WG and PRY (55).

### Structure-Activity Relationship of ACEi Peptides According to Source Proteins

The BLAST program was used for homology searches between manually obtained sequences and sequences in the Uniprot database. Analysis result indicated that a total of 27 rice proteins contained the H5 peptide sequence, while H6 had no identifiable source protein. H6 and H5 showed highly similar structure, differing in only one amino acid. Therefore, we speculated that H6 was a modified peptide generated during fermentation, while H5 corresponded to the source protein. To explore the structure-functional relationships, only H5 peptide that conformed to the rice protein sequence was selected to conduct further analysis.

Based on BLAST results, we determined that all source proteins belonged to *Oryza sativa* subsp. *japonica*, and most of them were glutelin. In addition, we found that the first amino acid linked to the N-terminus of H5 was Leu, and the amino acids connected to the C-terminus were His or Tyr. Thus, we synthesized three new peptides with increased terminal amino acid content.

Additionally, Pro, Trp, and Lys seem to be the most effective ones in increasing a peptide's ACEi potential (39). Between H5 and H6, the common sequence was identified as LIIP. Accordingly, new peptides with decreased terminal amino acids and exposed proline residues were also synthesized, and their IC_50_-values were measured (Table 5).

**Table 5:** IC_50_-values of synthetic peptides.

**Peptide**	**IC_**50**_ (μg/ml)**
LIIPQHH	1,206.67 ± 36.83
LIIPQHY	1,082.00 ± 38.85
LLIIPQH	829.00 ± 14.57
IIPQH	1,082.67 ± 36.83
LIIPQ	1,390.67 ± 21.40
LIIP	928.67 ± 24.01
H5 (LIIPQH)	143.33 ± 5.77
H6 (LIIPEH)	56.67 ± 12.02

Among LIIPQHH, LIIPQHY, and LLIIPQH, we found LLIIPQH possessed the highest ACEi capacity. This might be due to the fact that, among the added amino acids, Leu was the only hydrophobic residue, further emphasizing the importance of terminal hydrophobic amino acids for ACE inhibition. It has been suggested that amino acids with positive charge can promote ACE inhibition. However, despite containing two positively charged amino acids, LIIPQHH did not show the lowest IC_50_-value. In conclusion, the inclusion of positively charged amino acids improves inhibition rate only under certain conditions, rather than universally. The different IC_50_-values of LIIPQHH and LIIPQHY could attribute to the differences in the polarity of C-terminal residues, which might induce conformational changes in the peptide backbone that influence ACEi capacity.

For pruned peptides, IIPQH had a lower IC_50_-value than LIIPQ. which might be due to the positive charge of His that enhanced ACE inhibition. This is supported by a separated research, in which three of five ACE inhibitor peptides obtained from cherry seeds had a positively charge amino acid (H) in their C-terminal positions (58).

However, the activities of IIPQH and LIIPQ were not as high as that of LIIP. These differences might be a result of the C-terminal proline residues. C-terminal proline residues were found in lisinopril and enalapril peptides, suggesting that the presence of proline residues at the C-terminus was a specific feature associated with high ACEi activity and played a key role in ACE binding (38, 59). In addition, the imidazole ring of proline might easily interact with amino acid residues in the active center of ACE (60).

After the addition or subtraction of amino acids, the peptide sequence with the best activity was identified as LLIIPQH, followed by LIIP. However, all newly synthesized peptides exhibited lower ACEi activity than purified peptides obtained by us. Similar results had been found that some peptides showed high homology with ACEi peptides. Although these peptides shared the subsequence VTSTAV, not all of them exhibited significant ACEi effects (41).

## Conclusion

In this study, we obtained antihypertensive peptides from RWLE without enzymatic steps. Two novel peptides were purified and identified: LIIPQH and LIIPEH. They both showed strong ACEi activity and were classified as mixed-type inhibitors. Markedly, these two peptides showed great stability to ACE and digestive enzymes *in vitro*. Furthermore, we studied the structure-activity relationship of ACEi peptides by synthesizing similar peptides. Together, this study suggested that novel peptides with ACEi activity can be derived from rice wine lees and utilized to develop functional foods or antihypertensive medicine.

## Data Availability Statement

The original contributions presented in the study are included in the article/supplementary material, further inquiries can be directed to the corresponding authors.

## Author Contributions

ZH, GL, YC, and MS designed the experiment. ZH, GL, and ZQ conducted the activity evaluation experiments and collected and analyzed the data. ZH, GL, YC, and MS wrote and revised the manuscript. All authors contributed to the article and approved the submitted version.

## Funding

This work was financially supported by Guangdong Provincial Key Laboratory of Nutraceuticals and Functional Foods (2018B030322010), the Program for Guangdong Introducing Innovative and Entrepreneurial Teams (2019ZT08N291) China Postdoctoral Science Foundation (2020M672651), and Guangdong Province Modern Agricultural Industrial Technology System Innovation Team Project (2021KJ117).

## Conflict of Interest

The authors declare that the research was conducted in the absence of any commercial or financial relationships that could be construed as a potential conflict of interest.

## Publisher's Note

All claims expressed in this article are solely those of the authors and do not necessarily represent those of their affiliated organizations, or those of the publisher, the editors and the reviewers. Any product that may be evaluated in this article, or claim that may be made by its manufacturer, is not guaranteed or endorsed by the publisher.
